# Multifunctional Hybrid Composites with Enhanced Mechanical and Thermal Properties Based on Polybenzoxazine and Chopped Kevlar/Carbon Hybrid Fibers

**DOI:** 10.3390/polym10121308

**Published:** 2018-11-26

**Authors:** Hamid Abdelhafid Ghouti, Abdeldjalil Zegaoui, Mehdi Derradji, Wan-an Cai, Jun Wang, Wen-bin Liu, Abdul Qadeer Dayo

**Affiliations:** Institute of Composite Materials, Key Laboratory of Superlight Material and Surface Technology of Ministry of Education, College of Materials Science and Chemical Engineering, Harbin Engineering University, Harbin 150001, China; ham-ghouti@hotmail.com (H.A.G.); abdo@hrbeu.edu.cn (A.Z.); derradjimehdi1@gmail.com (M.D.); w.a.cai@hotmail.com (W.-a.C.); wj6267@sina.com (J.W.); abdul.qadeer@buitms.edu.pk (A.Q.D.)

**Keywords:** hybrid composites, Kevlar fibers, carbon fibers, bisphenol A-aniline-benzoxazine, mechanical properties, thermal properties

## Abstract

This work studied the structural, morphological, mechanical, and thermal properties of newly designed polymeric materials using high-performance hybrid fibers to reinforce the polybenzoxazine resins. To achieve this goal, hybrid fibers consisting of chopped Kevlar and carbon fibers were subjected to a silane surface treatment, incorporated into the resin matrix in various combinations, and then isothermally cured using the compression molding technique. The mechanical performances of the prepared composites were scrutinized in terms of bending and tensile tests. By way of illustration, the composites holding 20 wt % Kevlar fibers and 20 wt % carbon fibers accomplished a bending strength and modulus of 237.35 MPa and 7.80 GPa, respectively. Additionally, the same composites recorded a tensile stress and toughness of 77 MPa and 0.27 MPa, respectively, indicating an increase of about 234% and 32.8% when compared to the pristine resin’s properties. The thermogravimetric analysis denoted an excellent thermal resistance of the reinforced hybrid composites. Fourier transform infrared spectroscopy proved that the functional groups of the as-used coupling agent were effectively grafted on the external surfaces of the reinforcing systems, and further confirmed that the chemical reaction took place between the treated fibers and the polybenzoxazine matrix, although the scanning electron microscope showed a uniform dispersion and interfacial adhesion of the fibers within the resin matrix. In fact, the incorporation of treated fibers along with their good dispersion/adhesion could explain the progressive enhancement in terms of thermal and mechanical properties that were observed in the hybrid composites.

## 1. Introduction

Hybrid fibers, as reinforcing systems in polymer composites, have received much attention from researchers and scientists over the past few decades, since the rapid rise in the use of strong and rigid fibers—whether they are organic and/or inorganic fibers—has led to composite materials with enhanced properties that cannot be found in single typical phase materials. Usually, the reinforcing fibers used in the manufacture of polymer composites are basalt fibers (BFs), glass fibers (GFs), carbon fibers (CFs), and Kevlar fibers (KFs); of these fibers, GFs are the least expensive and are often used in a variety of functional fields such as in aviation and civil engineering [1]. Although CFs can be more or less expensive, they are copiously used in military and commercial manufacturing due to their advanced mechanical properties such as a high specific modulus and strength, and maintain their properties at relatively higher temperatures [2]. The development of CFs has experienced great acceleration in the attempt to reduce costs and maintain their properties, so many studies have been conducted to be able to adapt them to their commercial demand. It is in this context that manufacturers have introduced the use of short fibers instead of long fibers as Fu et al. demonstrated with a thorough study using short glass and carbon fiber reinforced polypropylene composites, concluding that the elongation of the composites fundamentally depended on the average fiber ratio, regardless of whether the modulus depended on the fiber volume factions [3]. In another study conducted on KFs as reinforcement for epoxy resin composites, the authors reported that these fibers considerably improved the abrasive properties of the filled composites [4]. Wan et al. performed a thorough investigation on 3D braided short Kevlar and carbon fibers reinforced bismaleimide hybrid composites, and reported that with a relatively high content of CF loadings as anticipated, all the carbon composites had higher flexural strengths than the corresponding KF composites [5]. In fact, it is certainly well known that KFs have good properties such as high tensile strength, stiffness, and very low density [6,7]. Nevertheless, they are originally quite brittle and their main shortcomings are their flexural and compression properties [7]. 

Generally, the manufacture of fiber reinforced composites induces much use of epoxy resins, polyester resins, and so on, but the former type of resin presents some drawbacks such as high shrinkage during curing and is very sensitive to water and temperature, without mentioning the necessary use of a coupling agent, which is costly for the manufacture of such polymers. To meet these challenges as above-mentioned, the use of bisphenol A-aniline-based benzoxazine (BA-a), as a new thermosetting resin that can be polymerized by the ring opening reaction without the use of kind of coupling agent and with no by-products resulting from its curing, has recently drawn much attention. With its near zero shrinkage, low flammability, high thermal stability, and high glass transition temperature [8,9,10,11,12,13,14], nevertheless, it should be understood that these thermosets have presented some defects such as their toughness deficiency, highly breakable characterization, and limited hardness for extremely exigent applications [15,16], which are significant disadvantages that researchers need to focus on [17,18]. To surmount the aforementioned shortcomings of the pure benzoxazine resins, several alternative strategies like the use of organic or inorganic fillers [19], and/or fibers as reinforcing systems, have been employed [20,21,22,23]. However, in order to further obtain new properties, it has been reported that hybridization is an adequate technique to achieve a multifunctional polymeric material that can surpass the cited limitations and the countless exigencies of the industry.

The motivation of the current research was to produce new hybrid materials using polybenzoxazine resins as polymer matrices and chopped Kevlar and carbon fibers as reinforcing systems. To achieve this objective, the hybrid fibers were initially treated with the silane coupling agent technique to minimize their agglomerations, and most importantly, promote better interactions within the resin matrix. Hereafter, various proportions of the as-modified fibers were incorporated into the benzoxazine and then cured using the typical hot press modeling method. Eventually, various experimental tests were carried out such as Fourier transform infrared spectroscopy (FTIR) and scanning electron microscopy (SEM) for the structural and morphological characterization, respectively. Mechanical tests in terms of the flexural and tensile tests were performed to explore the effects of the hybrid fiber incorporations on the mechanical properties of the prepared composites, while thermogravimetric analysis (TGA) was undertaken to evaluate the thermal stability of the materials. It is important to point out that this study could provide the scientific community with a new perception on using advanced hybrid composites in a panoply of interesting fields like aerospace, civil engineering, and military applications.

## 2. Materials and Experimental Study

### 2.1. Materials

A typical bisphenol A-aniline-based benzoxazine (BA-a) monomer was supplied by Jiangxi Huacui Advanced Materials Co., Ltd. (Fuzhou, China). The Kevlar fibers (KFs) and carbon fibers (CFs) were purchased from the DuPont Company (Shanghai, China) and Chuanjing Co., Ltd (Beijing, China), respectively. The physical and mechanical properties of the supplied fibers are summarized and given in Table 1. The 3-glycidyloxypropyltrimethoxy silane agent (GPTMS) with a molecular formula of C_9_H_20_O_5_Si was supplied by Union Silicon Chemical Co., Ltd (Nanjing, China), while ethanol (purity > 99.5%) was obtained from Aladdin Reagents Co., Ltd. (Shanghai, China). The distilled water used throughout this study was prepared in our laboratory.

### 2.2. Hybrid Composites Elaboration

**Figure 1:**
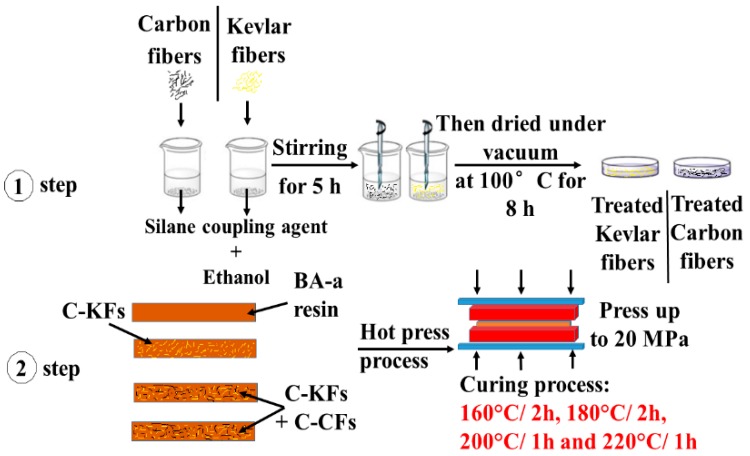
Preparation steps used for the elaboration of the hybrid composites.

The fabrication of the hybrid composites was as follows (see Figure 1): first, each fiber was subjected to a silane surface treatment using the GPTMS coupling agent. To achieve this, the as-received fibers were deposited in a receptacle containing the coupling agent (6% of filler) and ethanol, before being magnetically stirred for 5 h at room temperature. The collected fibers from the filtration process were then parched under a vacuum oven at 100 °C for 8 h. Meanwhile, the bisphenol A-and-aniline benzoxazine monomer was melted in a vacuum oven at 100 °C for 2 h, and then appropriate masses of the treated fibers were carefully added to the resin matrix as listed in Table 2, before manual stirring using a glass stirring rod. To ensure good diffusion of the reinforcing systems within the resin, the uncured composites were sonicated for 15 min, then the mixtures were poured into suitable molds and vacuumed under the temperature of 120 °C for 2 h to remove all of the air bubbles. Next, the mold was placed in a hot press and all of the prepared samples including the unfilled ones were isothermally cured by applying the pressure of 20 MPa and following the curing schedule: 160 °C for 2 h, 180 °C for 2 h, 200 °C for 1 h, and 220 °C for 1 h.

### 2.3. Experimental Study

The Fourier transform infrared spectroscopy (FTIR) test of the pure fibers, the treated fibers, and the fabricated specimens was recorded in the range of 4000–400 cm^−1^ using a Perkin-Elmer Spectrum 100 spectrometer (Waltham, MA, USA) fitted out with a deuterated triglycine sulfate detector and equipped with KBr optics at a resolution of 4 cm^−1^. The thermogravimetric analysis (TGA) of the fibers and the fabricated specimens was monitored on a TA Instruments Q50 (New Castle, DE, USA) at a rate of 20 °C/min, starting from 50 to 800 °C under a nitrogen (N_2_) atmosphere at flow rate of 50 mL/min. The flexural tests of all specimens were carried out using rectangular specimens with dimensions of 40 × 10 × 2 mm^3^ in an Instron 3365 instrument. The tensile tests for all of the manufactured composites were carried out at a crosshead speed of 1 mm/min using an Instron 5569 following the D3039/D3039M standard. A scanning electron microscope (SEM; Hitachi SUM800, Tokyo, Japan) was used to understand the state of the scattered fibers within the matrix, and to also demonstrate the fractured surface of the specimens after the mechanical tests.

## 3. Results and Discussion

### 3.1. Surface Characterization of KFs and CFs

**Figure 2:**
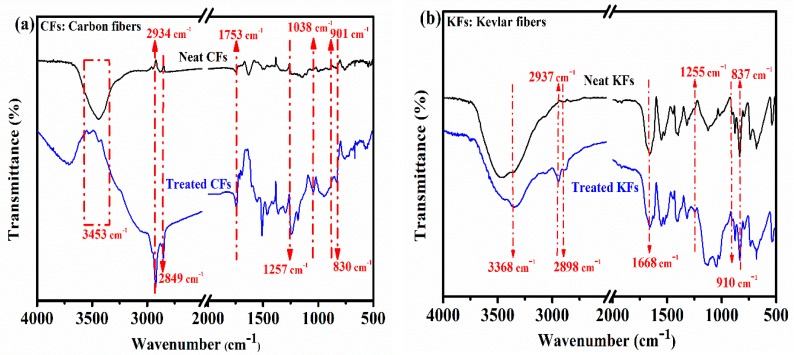
FTIR spectra of the treated and untreated carbon fibers (**a**) and Kevlar fibers (**b**).

As it is widely recognized as a useful technique that can be used to evaluate the chemical structures of the fibers before and after surface modification, FTIR analysis was conducted to prove whether the synthetic fibers were effectively coated with the functional groups of the as-used silane coupling agent. By carefully analyzing the data in Figure 2, it was found that both the pristine carbon and Kevlar fibers presented few functional peaks on their outer surfaces in the investigated range of 4000 to 400 cm^−1^. For instance, as shown in Figure 2a, the spectra of the neat CFs unfolded the presence of hydroxyl (‒OH) groups at 3453 cm^−1^; the frequencies detected at 2934 cm^−1^ and 2849 cm^−1^ were, respectively, attributed to the methylene group [24]; the non-intense band at 1753 cm^−1^ was assigned to the C=O vibrations, and the ranges of peaks in the frequency between 1600–1700 cm^−1^ were attributed to C=C as reported in previous studies [25]. All of these detected peaks through matching revealed the characteristics shown in the CF research [25]. However, the infrared spectrum of the treated CFs, as shown in Figure 2a, showed several functional peaks. Indeed, after the silane treatment, the silyl part appeared by showing the stretching vibrations of CH_2_ groups in the frequencies of 2934 cm^−1^ and 2849 cm^−1^. The detected peaks at 1257 cm^−1^ and 901 cm^−1^ could be attributed to the stretching vibrations of siloxanes and epoxy groups [26], respectively, while the band seen at 830 cm^-1^ could be due to the C–O stretching vibrations. The IR spectrum of the pure Kevlar fibers unfolded the N–H groups at 3368 cm^−1^ and 1560 cm^−1^, and showed less in intensity for the treated ones; this could be interpreted by the partial substitution of N–H group of treated KFs [27]. Additionally, a further peak appeared at 2937 cm^−1^, showing the C–H vibration of the methylene group [28], the small peak of 1255 cm^−1^ was assigned to the Si–O–Si groups, and the epoxy peak appeared at 910 cm^−1^. Based on the FTIR results, we can say that the included silane groups were effectively transplanted on the outer surfaces of the synthetic fibers (i.e., CFs and KFs).

### 3.2. FTIR Studies of the Specimens

**Figure 3:**
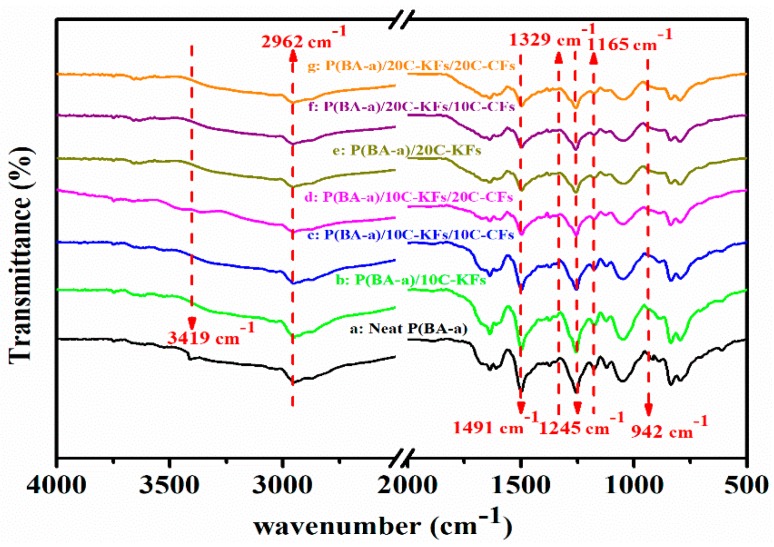
FTIR of the hybrid composites with different hybrid fiber contents.

Figure 3 presents the FTIR results of the pristine P(BA-a) resin and its relative composites with different fiber contents. Based on the spectra of Figure 3a, we can say that the characteristic bands of the unfilled P(BA-a) resins were perceptible. As an example, the typical stretching vibration peak of the methylene group was observed at around 2962 cm^−1^ [29], and the band at 942 cm^−1^ was assigned to the phenol groups linked to the oxazine ring; this denoted the presence of a benzoxazine group [30]. At the 1329 cm^−1^ and 11,645 cm^−1^ absorptions, the CH_2_ wagging and asymmetric stretching modes of C−N−C were found, respectively. Furthermore, a tri-substituted benzene ring was observed at the transmittance band of 1491 cm^−1^. Meanwhile, the spectrum of the hybrid composites, given in the inset of Figure 3b–g, showed variable intensities and some peaks, like the bands at 1491 cm^−1^ and 942 cm^−1^, disappeared, while a new peak occurred at 3419 cm^−1^, which could correspond to the hydrogen bonding between the resin and the hybrid fibers, and this could further explain the enhanced interaction between the fibers and the resin [29]. Supported by the FTIR analysis, we can infer that the silane modified fibers reacted with the polymer matrix.

### 3.3. Thermal Properties

**Figure 4:**
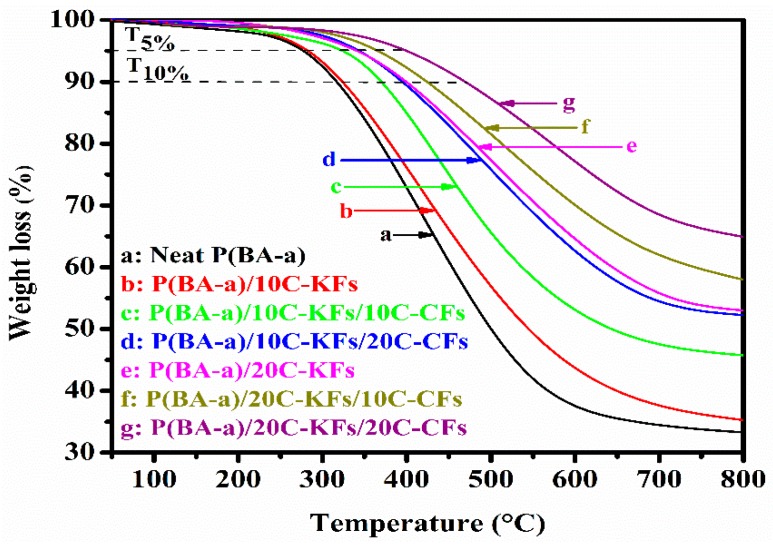
Thermal resistance of the fabricated composites with different hybrid fiber loadings.

The enhancement of thermal stability is a primary objective for meeting the challenges that many polymer composites may encounter. In fact, TGA is a technical test where the weight loss of a substance is examined as a function of temperature under a controlled atmosphere. This current section studied the thermal behavior of the hybrid composites under a nitrogen atmosphere as depicted in Table 3 and Figure 4, which show the thermal stability parameters and their corresponding TGA curves, respectively.

**Figure 5:**
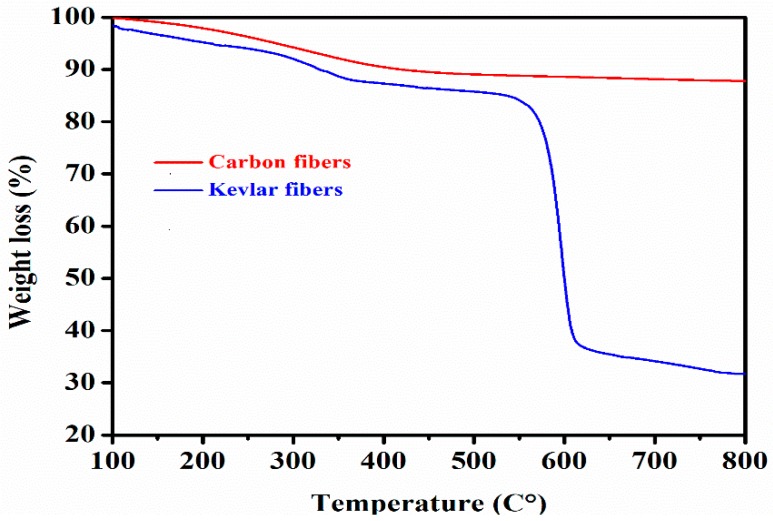
Thermal stability of the carbon fibers and Kevlar fibers.

From our initial observations, it was noticeable that the decomposition process of the pure resins and their subsequent composites exhibited one thermal step degradation. Furthermore, for all of the specimens with fiber reinforced P(BA-a) composites, as recorded in Table 3, we could observe that the values of *T*_5%_ (the weight loss at 5%), *T*_10%_ (the weight loss at 10%), and *T*_HRI_ (the heat resistance index) increased and were appreciably higher than those of the pure resins properties. However, the char yield at 800 °C gradually increased, demonstrating the presence of more moisture in the composites. This could mostly be due to the high-performance temperature of the hybrid fibers, which offer thermal synergic effects (also designated as hybrid effects) to the poly(BA-a) resins. As depicted in Table 3, the starting thermal degradations of poly(BA-a) in terms of *T*_5%_ and *T*_10%_ were 277 °C and 317 °C, respectively, which could be due to the evaporation of macromolecular groups such as the amine and phenolic groups [31,32]. Meanwhile, the hybrid composites exhibited much higher initial degradations. For instance, within all of these samples, the hybrid composites filled with 20% KFs and 20% CFs exhibited the highest values in terms of *T*_5%_, *T*_10%_, *T*_HRI_, and the char yield was found to be higher by 64.9% than those of the neat P(BA-a) resins. The enhancement seen in the thermal stabilities of the hybrid composites could be due to the excellent dispersion of the hybrid fibers within the resin matrix. Additionally, the high reaction interaction between the intra hydrogen bonding of the free hydroxyl groups of Poly(BA-a) with the silane treated hybrid fibers created more density and allowed the fibers to be used as thermal shielding, which made the evaporation of the macromolecules more difficult to attempt [33]. Furthermore, to explain the thermal enhancement of the hybrid composites, the thermal stability of CFs and KFs was also carried out by the TGA to obtain a clear indication about their roles in providing excellent thermal properties of the hybrid composites. As reported in Figure 5, the CFs showed good thermal stability when compared to the Kevlar fiber properties, this could be related to the microstructural properties of CFs, which, as their name indicates, mainly contain carbon, and the presence of graphite in their structure is known for its high thermal resistance [34]. Effectively, the weight losses at 5% and 10% were at 280 °C and 418 °C, respectively, with no major weight loss up to 800 °C for the CFs. However, the weight loss of the KFs at 5% and 10% were 190° C and 313 °C, respectively, with the char yield of 31%% at 800 °C. We can conclude that the main factor enhancing the thermal stability was the CFs, with less influence from the KFs.

### 3.4. Bending Test

**Figure 6:**
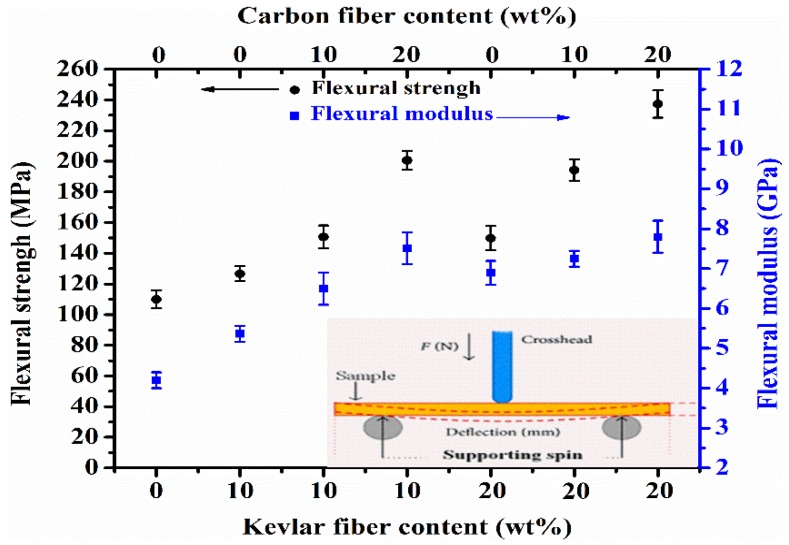
Bending properties of the hybrid benzoxazine composites.

The bending test was carried out for all the studied specimens, and the obtained curves with their corresponding results (i.e., flexural strength and flexural modulus) are illustrated in Figure 6 and tabulated in Table 4, respectively. Interestingly, all the composites unfolded good flexural strength and modulus values. For instance, as presented in Table 4, the flexural strength of the composites containing 10 wt % and 20 wt % KFs was recorded to be 126 MPa and 149 MPa, respectively, which were evidently higher than that of the reference resin with 110 MPa. This improvement was expected since it is known that loading high mechanical performance fibers with such a good adhesion/dispersion can raise the mechanical properties of the polymer composites. In the meanwhile, adding appropriate masses of CFs to the KFs filled poly(BA-a) effectively increased the flexural strength and modulus up to 237 MPa and 7.8 GPa, respectively, which gave an outstanding enhancement of 115% and 195% over the free P(BA-a) resin parameters. Indeed, as illustrated in the hybrid composites containing 20% KFs and 20% CFs, it exhibited the highest flexural strength and modulus, this confirmed that the KFs and CFs remained playing the major role in the bending properties of the composites. Although, we could not ignore that the high flexural parameters of the randomly oriented KFs and CFs in the resin and their good dispersion and adhesion could afford the desired stiffness and inflexibility by soaking up the energy applied in the bending test [35]. Furthermore, another explanation that could be attributed to this enhancement is the possibility of the hybridization to occupy more vacant point around the poly(BA-a) resin surfaces to cover each weak spot against mechanical stress that resulted in reducing all the released space of the Poly(BA-a) resin. A similar deduction for basalt fibers reinforced polymer composites were also reported [36,37].

### 3.5. Tensile Test

**Figure 7:**
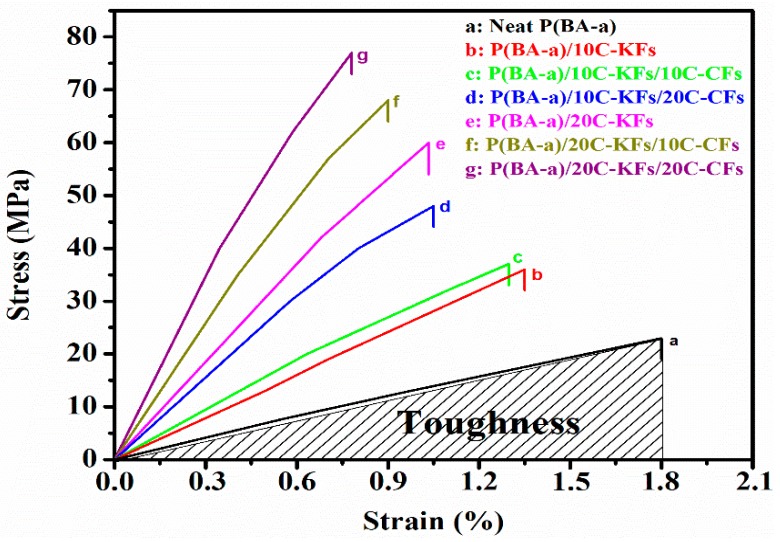
Tensile stress/strain curves of the P(BA-a)/C-KFs/C-CFs composites.

The tensile test was done for all the specimens to study their tensile properties as reported in Figure 7. As the amount of the hybrid contents increased, the stress of the composites grew, showing a remarkable enhancement while all composites presented a slight decrease in tensile strain as the ratio of KFs and CFs increased. The best tensile performance was shown on the P(BA-a)/20C-KFs/20C-CFs reporting their tensile stress with 77 ± 2.24 MPa, which was a performance value of 220% over the pristine resin. Moreover, the strain dropped as the hybrid content was raised, reaching its lowest value of 0.76% for the same previously cited composite, and this could explain that for the chopped fibers not enough rigidity was offered to the resin. It could be seen that the tensile data for the chopped KFs and CFs hybrids were quite similar to the bending results. In fact, it has been shown that the fibers had a great task in the tensile load carrying capacity of the structure.

**Figure 8:**
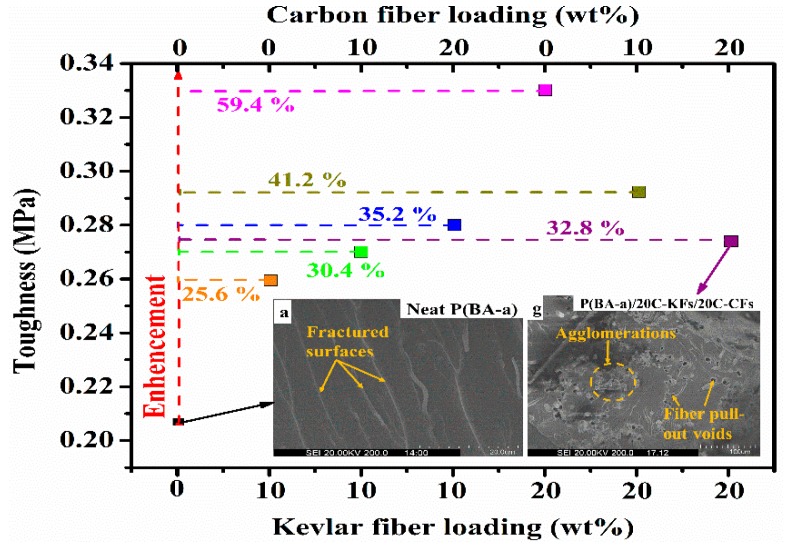
Toughness curve of the P(BA-a)/C–KFs/C–CFs composites.

Meanwhile, the chopped fibers were randomly oriented within the benzoxazine resin matrix, which could explain their high mechanical performances in the multitude direction. To consolidate this approach, the toughness values, which are expressed by the total energy absorption of the materials just before the rupture, were computed by integrating the area under the tensile stress/strain curves [38,39]. In this context, the toughness investigation depicted in Figure 8 did show that the hybrid composites were tougher when more hybrid contents were added, but the toughness enhancement did not have the same tendency. Nevertheless, even reaching the highest content of the hybrid fibers with 20% KFs and 20% CFs, the hybrid composites were still tougher than the pristine ones. From this, we can conclude that there was a strong relationship between the fiber loading and the toughness state of the composites. These enhancements seen in the toughness behavior could be explained by the fact that the silane coupling agent might form a stable hindrance layer between the hybrid fibers, which resulted in the inhibition of their agglomeration process, and hence enhanced their dispersion and interfacial adhesion within the resin matrix. Note that the exceptional features of the reinforcing fibers may surely take part in these improvements through their high abilities to transfer the mechanical loads. Regarding these results and with respect to the neat P(BA-a), all the other specimens showed remarkable improvements in their mechanical properties.

### 3.6. SEM Analysis

**Figure 9:**
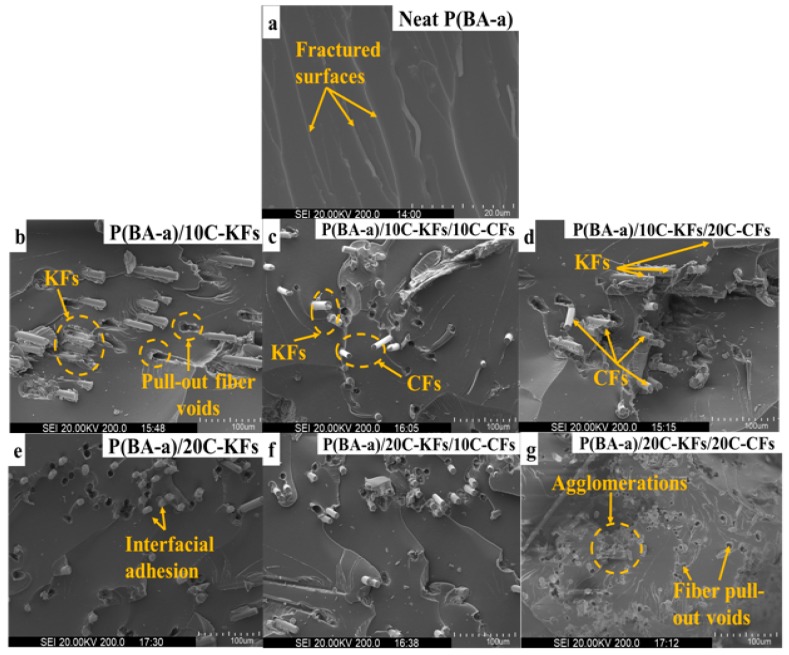
SEM micrographs of the hybrid benzoxazine composites.

The SEM images show what the states of dispersion, adhesion, and interfacial fracture were like after carrying out the mechanical test within the polymer matrix. The fissured areas of the neat Poly(BA-a) and their derivative composites at different amounts of fiber loadings are shown in Figure 9. Evidently, the native resin possessed a uniform and continual form of small waves on the unwrinkled and plane surface, which means that there were many crack propagations generated by the mechanical test expressing the high breakability and brittleness as seen in Figure 9a. This observation can mean that there were no obstacles to overcome and/or to slow down the crack spread. On the other hand, the SEM images, displayed in Figure 9b–g, for the composites reinforced with KFs and the hybrid fibers, showed a collapsed fracture on the matrix resin. Moreover, the randomly oriented fibers were found to be well dispersed over all sections of the composites. Additionally, we noticed that each fiber was sheltered by a linking layer of the poly(BA-a) resin, which demonstrated the excellent adhesion between the fibers and the resin matrix, and confirmed once more the enhancement seen in the mechanical and thermal properties. However, we observed some holes due to the grabbing produced by the mechanical force, which resulted from fiber/matrix interfacial debonding and fiber pulled out voids. The debonding mechanism of the fibers within the matrix could allow the composites to consume a higher fracture energy ensuring an effective toughness which put forward that the adhesion was systematically enhanced [24,40]. In summary, the good results observed in the micrographs of the reinforced composites could be explained by the fact that treating the hybrid fibers with the silane coupling agent effectively improved the roughness of the composites by replacing the observed fracture in the reference resin with a multitude of micro-cracks.

## 4. Conclusions

To sum up, this study focused on comprehending the positive effects of short hybrid Kevlar fiber and carbon fiber loading reinforcements on the thermal, mechanical, structural, and morphological properties of the bisphenol A-aniline-based benzoxazine resin. The mechanical properties via the bending and tensile performances were remarkably improved by the high performance hybridization. Meanwhile, the starting thermal degradation supported by the TGA tests provided excellent results regarding the pristine resin. Note that these efficient improvements were due to the further loading of CFs upon KF reinforced hybrid composites. For instance, composites with 20% KFs and 20% CFs showed the best mechanical and thermal improvements. Indeed, the bending strength, bending modulus, tensile stress, *T*_5%_, *T*_10%_, *T*_HRI_, and *Y*c at 800 °C were recorded as 237.35 MPa, 7.80 GPa, 77 MPa, 400 °C, 471 °C, 275 °C, and 64.9%, respectively. The SEM micrographs further demonstrated the uniform dispersion and interfacial enhancement between the hybrid fibers and the polymer resin. We believe that the advancements seen in the studied properties are due to either the excellent performances of the hybrid KFs and CFs fibers, their excellent scattering around the surface as well as their interfacial adhesion within the polymer resin.

## Figures and Tables

**Table 1:** Physical and mechanical properties of the Kevlar (KFs) and carbon fibers (CFs).

Fibers	Diameter (µm)	Length (mm)	Density (g/cm^3^)	Tensile Strength (MPa)	Young’s Modulus (GPa)
Kevlar fiber	12	6	1.45	2860	67
Carbon fiber	7	6.2	1.76	3500	260

**Table 2:** Neat and hybrid formulations investigated with different weight ratios.

No.	Material Type	BA-a (wt %)	KFs (wt %)	CFs (wt %)
1	Neat P(BA-a)	100	0	0
2	P(BA-a)/10C-KFs	90	10	0
3	P(BA-a)/10C-KFs/10C-CFs	80	10	10
4	P(BA-a)/10C-KFs/20C-CFs	70	10	20
5	P(BA-a)/20C-KFs	80	20	0
6	P(BA-a)/20C-KFs/10C-CFs	70	20	10
7	P(BA-a)/20C-KFs/20C-CFs	60	20	20

P(BA-a): Polybenzoxazine. BA-a: Bisphenol A aniline based benzoxazine resin. C-KFs: Chopped Kevlar fibers. C-CFs: Chopped carbon fibers.

**Table 3:** Thermal stability parameters of the hybrid composites.

No.	Material Type	*T*_5%_ (°C)	*T*_10%_ (°C)	*Y*_c_ (%) at 800 °C	*T*_HRI_ (°C)
1	Neat P(BA-a)	277	317	33.2	175
2	P(BA-a)/10C-KFs	286	326	35.2	182
3	P(BA-a)/10C-KFs/10C-CFs	326	373	45.8	203
4	P(BA-a)/10C-KFs/20C-CFs	346	393	52.2	225
5	P(BA-a)/20C-KFs	332	398	53.7	227
6	P(BA-a)/20C-KFs/10C-CFs	366	424	58.8	246
7	P(BA-a)/20C-KFs/20C-CFs	400	471	64.9	275

*T*_HRI_ (°C) = 0.49 × [*T*_5%_ + (*T*_30%_ **−** *T*_5%_)].

**Table 4:** Bending test results of the neat benzoxazine and the hybrid composites.

No.	Material Type	Flexural Strength (MPa)	Flexural Modulus (GPa)
1	Neat P(BA-a)	110 ± 3.3	4.20 ± 1.3
2	P(BA-a)/10C-KFs	126.65 ± 2.4	5.36 ± 1.4
3	P(BA-a)/10C-KFs/10C-CFs	150.58 ± 5.1	6.49 ± 5.8
4	P(BA-a)/10C-KFs/20C-CFs	221.60 ± 4.2	7.51 ± 3.4
5	P(BA-a)/20C-KFs	149.78 ± 7.4	6.89 ± 5.3
6	P(BA-a)/20C-KFs/10C-CFs	194.26 ± 6.8	7.24 ± 1.2
7	P(BA-a)/20C-KFs/20C-CFs	237.35 ± 7.9	7.80 ± 5.4
